# Chimeric receptor-binding domain vaccine design and sequential immunization enhanced broadly neutralizing antibody responses against COVID-19

**DOI:** 10.3389/fimmu.2025.1543212

**Published:** 2025-03-27

**Authors:** Xiao Yang, Xin Tang, Ying Sun, Hualong Xi, Wei Peng, Lu Yan, Wenjing Teng, Yang Zang, Chunlai Jiang

**Affiliations:** ^1^ Research and Development Department, Changchun BCHT Biotechnology, Changchun, Jilin, China; ^2^ National Engineering Laboratory for AIDS Vaccine, School of Life Sciences, Jilin University, Changchun, Jilin, China; ^3^ Research and Development Department, The Medium Therapeutics Co., Ltd., Suzhou, Jiangsu, China

**Keywords:** SARS-CoV-2, broadly neutralizing antibody, variant of concern, receptor-binding domain, Fc fragment

## Abstract

**Introduction:**

Vaccines developed using modified messenger RNA (mRNA) technology show robust efficacy against severe acute respiratory syndrome coronavirus 2 (SARS-CoV-2) infection in humans. However, viral evolution in human and non-human hosts may compromise vaccine performance due to the emergence of new variants with strong immune-escape abilities. Therefore, a coronavirus disease 2019 (COVID-19) vaccine that induces high levels of broadly neutralizing antibodies (bnAbs) and responds quickly to viral mutations is urgently required.

**Methods:**

Here, we designed a bivalent mRNA vaccine, RBDco, based on the variant of concern (VOC) spike (S) protein receptor-binding domain (RBD) chimeric from different lineages fused with Fc fragments.

**Results:**

In mice and non-human primates, RBDco effectively induced neutralizing antibodies against several pseudoviruses, including the possible epidemic variants XBB.1, XBB.1.9.1, and EA.1 pseudoviruses. In mice, RBDco induced bnAbs against 11 SARS-CoV-2 variant pseudoviruses from different lineages. The neutralizing antibody titers against the prototype D614G and the epidemic variant XBB.1.16 were 19666 and 13274, respectively. RBDco induced mice secrete interferon-γ (IFN-γ) under the stimulation of RBD proteins of SARS-CoV-2 variants. In the mouse challenge model, RBDco treatment led to a 10-fold reduction in the viral load in the lungs of mice after the challenge. These results suggest that RBDco can induce a bnAb response and cellular immune response in animals, thereby preventing the occurrence of COVID-19. Furthermore, the sequential immunization results showed an improved neutralizing antibody titer in RBDco-boosted groups relative to the inactivated group. Enhanced differentiation of memory T cells was observed in the RBDco-boosted group.

**Discussion:**

Overall, RBDco can induce bnAbs in animals via chimeric RBDs with the SARS-CoV-2 VOC in different lineages and is a candidate for mRNA vaccine for a rapid response to viral mutations.

## 1 Introduction

The coronavirus disease 2019 (COVID-19) outbreak, caused by severe acute respiratory syndrome coronavirus 2 (SARS-CoV-2), has reshaped human life worldwide. Since the outbreak of the pandemic, prevention and control strategies have shifted from closed control to transmission prevention strategies and social group immunity. COVID-19 has gradually become an epidemic that will likely persist alongside human development for the foreseeable future. Vaccines are currently the most promising strategy to curb the spread of the epidemic. The COVID-19 vaccine was launched in 2020. The mRNA-1273 (Moderna) and BNT162b2 (BioNTech and Pfizer) vaccines were developed soon after the onset of the COVID-19 pandemic, demonstrating over 90% protective efficacy during the initial stages of the pandemic (1, 2). However, quickly emerging SARS-CoV-2 variants, such as Delta and Omicron, have substantially reduced the protective efficacy of vaccines through mutations in the spike (S) protein (3, 4). To cope with the rapidly mutating virus, an effective COVID-19 vaccine that can induce broadly neutralizing antibodies (bnAbs) against diverse variants, especially the Omicron variant, is urgently required (5–7).

The S protein of SARS-CoV-2 forms a trimer on the surface of the virus and plays pivotal roles in viral binding, fusion, and entry (8). Initial studies on COVID-19 vaccines identified the gene encoding the SARS-CoV-2 S protein (9–11). The receptor-binding domain (RBD) is the major target of neutralizing antibodies (nAbs) and a promising antigen for vaccine development owing to its immunodominance (12, 13). For instance, the ZF2001 subunit vaccine reported by Gao et al. used chimeric RBD dimers combining two heterologous RBDs. These chimeric vaccines elicited broader serum neutralization and conferred better protection in mice (14). Notably, the Delta–Omicron chimeric RBD dimer vaccine elicited superior protection against challenges by either Delta or Omicron SARS-CoV-2 in mice (15). Thus, potential vaccine candidates may contain chimeric RBDs of different lineages of variants of concern (VOCs).

Rational vaccines are also needed to elicit long-term antibodies in humans, provide prolonged protection against SARS-CoV-2 infection, and respond to future COVID-19 pandemics. Jiang et al. reported a candidate vaccine, RBD-Fc, consisting of RBD and Fc fragments of human IgG. The Fc fragment functioned as an immunopotentiator, enhancing the immunogenicity of RBD (16–18). Additionally, to extend the half-life of antibodies *in vivo* and increase the affinity for IgG Fc binding to the neonatal Fc receptor (FcRn), incorporating the YTE mutation may be a rational choice in vaccine design. In a phase I clinical study, the YTE-mutated antibody could increase the half-life of antibodies by 4–5-fold in patients (19). Based on these studies, we believe that chimeric RBD vaccines with an Fc fusion have the potential to induce higher bnAb responses.

Messenger RNA (mRNA) technology, commonly known as the “master key,” is a cutting-edge technology in the global biopharmaceutical field, rated as one of the top ten innovative pharmaceutical technologies worldwide in 2019 (20). During the fight against the COVID-19 pandemic, mRNA vaccines utilized lipid nanoparticle (LNP) delivery systems and achieved substantial success. The mRNA vaccine platform has unique advantages, such as a swift pandemic-response strategy owing to its flexibility in immunogen design and its potential for rapid and scalable manufacturing, mainly because of the high yields from *in vitro* transcription reactions (21, 22).

In this study, we designed a chimeric mRNA vaccine candidate, RBDco, which contained RBDs of different lineages of VOC. Additionally, we included a human IgG Fc fragment with a YTE mutation to enhance the half-life of antibodies. The immunogenicity and protective effects of RBDco against epidemic viruses were then evaluated in both mice and non-human primates. Given the widespread usage of inactivated vaccines, we also evaluated the potential application of our vaccine as a heterologous booster shot. This strategy is expected to offer improved responses to more rapid viral mutations by replacing the chimeric RBD sequence, thus becoming an alternative to the COVID-19 vaccine.

## 2 Materials and methods

### 2.1 Facility and ethics statement

All experiments with live SARS-CoV-2 were performed in enhanced biosafety level 3 (P3+) facilities. All experiments involving mice and non-human primates were performed in accordance with the regulations outlined in the Guide for the Care and Use of Laboratory Animals of the Ministry of Science and Technology of the People’s Republic of China. All animal experiments were strictly implemented in accordance with biosafety operating procedures and animal ethics requirements, and humane care was provided to animals to ensure their welfare. All experimental procedures were conducted under anesthesia, which met the requirements of biological safety and animal ethics. All animal experiments were reviewed and approved by the Experimental Animal Ethics Committee of the Institute of Medical Biology, Chinese Academy of Medical Sciences (DWSP202310003).

### 2.2 mRNA synthesis and purification

**Figure 1:**
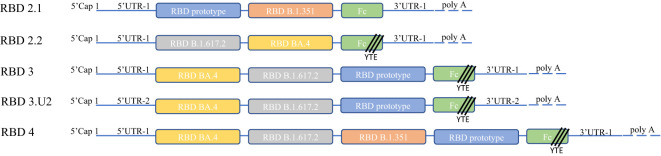
Molecular design and construction. We optimized 5′-UTR-2 in the RBD3U2 construct to express proteins at high levels. All constructs carried Fc fragments fused at the ends of RBDs to achieve high immunogenicity and persistence. YTE (Met252Tyr, Ser254Thr, and Thr256Glu) mutations were incorporated in the Fc fragments of RBD2.2U1, RBD3U1, RBD3U2, and RBD4U1 to prolong the half-life of the expressed proteins. The Fc fragment was connected to the RBD using a G4S linker. The main body of the construct was composed of a chimeric VOC RBD, including the RBD prototypes (RBD2.1U1, RBD3U1, RBD3U2, and RBD4U1), RBD B.1.351 (RBD2.2U1 and RBD4U1), RBD B.1.617.2 (RBD2.2U1, RBD3U1, RBD3U2, and RBD4U1), and RBD BA. 4 (RBD2.2U1, RBD3U1, RBD3U2, and RBD4U1).

**Figure 2:**
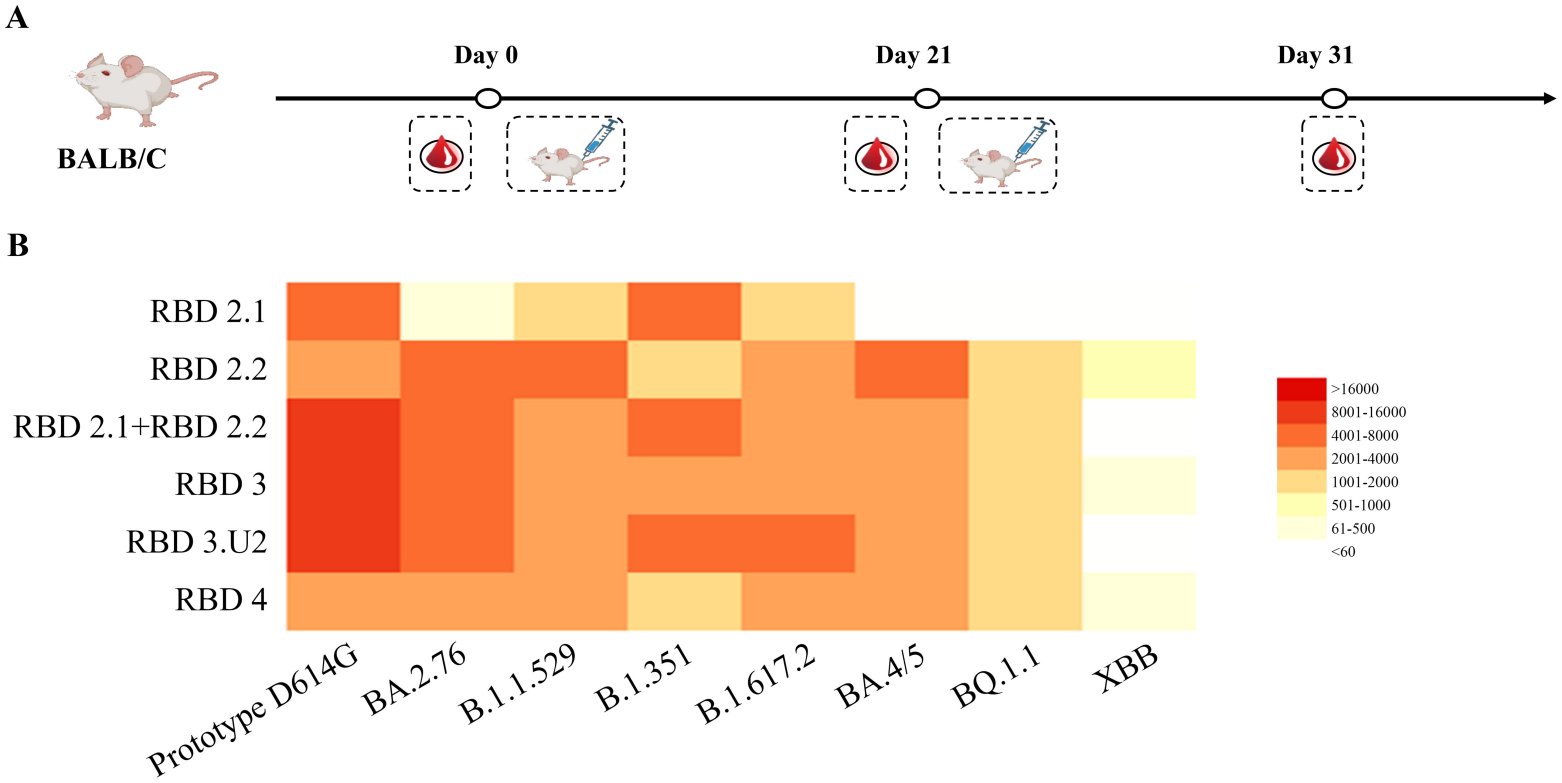
Comparing the neutralization breadth and potency among different immunogens. **(A)** Vaccination regimen for mice. Mice (n = 6) were randomly assigned to six groups and immunized with the respective immunogens twice at 3-week intervals. **(B)** Analysis of neutralization activities in mouse sera. Sera were collected 10 d after the final immunization, and neutralization was performed against the virus panel. The 50% inhibitory doses (ID_50_) against pseudoviruses were analyzed using a heatmap. The neutralization potency is depicted using a color gradient, as indicated in the histogram.

All mRNAs are illustrated in **Figures 1**, **2**. Each mRNA strand comprised a 5′ Cap1 structure, 5′-untranslated region (UTR), coding sequence, 3′-UTR structure, and a polyA tail. mRNAs were transcribed using T7 RNA polymerase (T7 RNA Transcription Enzyme Mix, GMP-E131-M001, Novoprotein, Beijing, China) according to the manufacturer’s instructions. Briefly, 50 µg/mL linear plasmid was used as template for an IVT reaction at 37°C for 6 h with ribonucleotides (10 mM ATP, 10 mM GTP, 10 mM CTP, and 10 mM N1-Me-UTP; Glycogene, China), 2000 U RNase Inhibitor (RNI-ME001-C, Glycogene), and 50 µg PPase (PYR-EE201-B, Glycogene). The cap analog (3-OMe-GAG, CA-1006, Glycogene) was co-transcribed into mRNA strands. The IVT reaction was quenched by adding 100 units of DNase I (GMP-E127, Novoprotein). All mRNAs were purified using the oligo-dT affinity method for subsequent detection.

### 2.3 Preparation and characterization of mRNA-loaded LNPs

mRNA-loaded LNPs were prepared using a microfluidic method. Briefly, the mRNA was dissolved in RNase-free citrate buffer (50 mM, pH 4.0). Lipids, including [(4-hydroxybutyl) azanediyl] bis (hexane-6,1-diyl) bis (2-hexyldecanoate), DSPC, cholesterol, and methoxypoly (ethylene glycol)-ditetradecylacetamide were dissolved in anhydrous ethanol at a molar ratio of 46.8:8.9:42.7:1.6. The lipid solution was then mixed with the mRNA solution at a 1:3 ratio using a microfluidic device and further diluted with Tris buffer. The diluted solution was then transferred to an ultrafiltration tube. After diafiltration and sterile filtration, the final product was stored at -20°C. The average diameter and PDI were measured using dynamic light scattering (Malvern Zetasizer Ultra, UK). The mRNA content and encapsulation efficiency were determined using the RiboGreen assay (Thermo Fisher Scientific, USA) following the manufacturer’s instructions.

### 2.4 Target antigen expression

HEK293 cells were seeded at a density of 10^6^ cells/well in 6-well plates and transfected with mRNA (2 µg/well) using Lipofectamine 3000 according to the manufacturer’s instructions. Cells and supernatants were collected 48 h after transfection. Cells were lysed using cell lysis buffer (Cat#: 9803S) and then centrifuged at 10 000 × *g* for 15 min at 4°C to collect the cell lysates. The cell lysates and supernatants were loaded and separated on NuPAGE^®^ 4–12% Bis-Tris Gels (NP 0336BOX, Thermo Fisher Scientific) and transferred to polyvinylidene difluoride membranes. Immunoblotting was performed using an anti-nCov-spike-RBD Ab (40150-R007, 1:1000, Sino Biological, China). Anti-rabbit IgG-HRP-linked antibody (7074S, 1:500, CST) and anti-mouse IgG, HRP-linked antibody (7076S, 1:2000, CST) were used as secondary antibodies. Fc fragments were detected via western blotting using goat anti-human IgG Fc (ab2098, 1:5000, Abcam, USA) and horse radish peroxidase (HRP)-linked antibody (ab97225, 1:2000, Abcam, USA).

### 2.5 Immunization and challenge

Prior to vaccination, the vaccines were thawed at 4°C for 30 min and diluted to the desired concentrations using saline. For mouse vaccination, groups (n = 6) of 6–8-week-old female BALB/c mice were anesthetized with intraperitoneal tribromoethanol and then intramuscularly immunized with 100 μL of 1.25, 2.5, or 5 μg vaccine candidates or control. Three weeks later, a second dose was administered to boost the immune responses. For the mouse challenge model, Chen et al. report that These emerging variants of SARS-CoV-2 Can infect standard laboratory mice after intranasal instillation (23), so we chose standard laboratory BALB/c mice as model. Mice were anesthetized with intraperitoneal tribromoethanol and then challenged with Omicron (XBB. 1.16; virus batch number 23V08P, original titer of 1 × 10^7^ TCID50/mL) via nasal drip (100 μL/mouse), at a dose of 1 × 10^6^ TCID50 per mouse. Mice were monitored daily for clinical signs and weight loss, with 70% of their initial weight considered a humane endpoint. After the challenge, mice were anesthetized using gas inhalation at 1, 2, 3, 4, and 5 dpi, nasal and pharyngeal swabs were collected and lysed with 800 μL TRIzol, and 200 μL of the RNA template was extracted from the lysate using an automatic nucleic acid extractor. Quantitative RT PCR (one-step method) was then performed. On the fifth day after the challenge, mice were euthanized, and their lungs were dissected to observe general pathological changes. The left lung lobe was fixed and sliced for pathological examination using HE staining, whereas the right lung lobe, nasal turbinate, and tracheal tissue were used for viral load evaluation. RNA from the lungs was used in qRT-PCR assays (one-step method) to detect the viral load. At the endpoint of the experiments, mice were euthanized by cervical dislocation. For the vaccination of non-human primates, groups (n = 4 half male and half female) of 3-7 years old crab-eating macaques were intramuscularly immunized with 25 μg vaccine candidates twice, on days 0 and 21, and detected the levels of binding and neutralizing antibodies against different SARS-CoV-2 strains in their sera on days 36 and 64. Sera samples from mice or cynomolgus monkeys were collected and inactivated at 56°C for 0.5 h before measurements of SARS-CoV-2-specific IgG and neutralizing antibodies.

### 2.6 Viral load determination

Real-time quantitative PCR (qRT-PCR) was performed to detect viral genomic RNA. The primers and probe used to detect the N gene of the viral genome were as follows: N Forward, 5′-GACCCCAAAATCAGCGAAAT-3′; N Reverse, 5′-TCTGGTTACTGCCAGTTGAATCTG-3; N-probe, 5’-FAM-ACNCCGCATTACGTTTGGTGGACC-BHQ1-3’. These primers were designed and validated by the Institute of Medical Biology, Chinese Academy of Medical Sciences. SARS-CoV-2 viral loads were expressed on a log10 scale as viral copies per gram after calculation using a standard curve. RNA was extracted from the lungs and nasal turbinates using Direct-zol RNA Miniprep Kits (Zymo Research, Tustin, CA, USA). qRT-PCR was performed using the TaqMan^®^ Fast Virus 1-Step Master Mix (Thermo Fisher Scientific, Waltham, MA, USA).

### 2.7 Pseudovirus-based neutralization assay

The neutralization assay was conducted using pseudoviruses. For pseudovirus production, 293T cells were co-transfected with three plasmids, namely pMD2.G, pSPAX2, and an expression plasmid encoding the codon-optimized full-length spike protein derived from different mutant SARS-CoV-2 strains. These plasmids can activate a lentiviral packaging system with the expression of TMPRSS2. Briefly, 5 × 10^6^ 293T cells were seeded in a T75 flask 1 d prior to transfection. On the following day, cells were co-transfected with 4 μg of each of the three plasmids. Pseudoviruses were harvested from the culture supernatant at 72 h post-transfection and subsequently stored at -80°C until further use. Sera samples obtained from immunized animals were serially diluted thrice with cell culture medium and incubated with the pseudovirus suspension at a 1:1 ratio in 96-well plates for 1 h at 25°C. Subsequently, Opti-HEK293/ACE2 cells were added to the serum-virus mixture, and the plates were incubated at 37°C and 5% CO_2_ for 48 h. The degree of SARS-CoV-2 pseudoviral infection was assessed by measuring the luciferase activity using the luciferase assay kit (PerkinElmer, MA, USA). The half-maximal inhibitory concentration (IC_50_) was determined as the highest serum dilution that inhibited pseudoviral infection by over 50% relative to that of the control group. Non-linear regression analysis was conducted using GraphPad Prism software version 9.00 (San Diego, CA, USA) with the four-parameter logistic sigmoidal dose-response model to calculate the IC_50_ value.

### 2.8 IFN-γ and IL-4 enzyme-linked immunospot (ELISpot) assays

The number of spleen cells secreting IFN-γ and IL-4 was detected using ELISpot assays. Briefly, single-cell suspensions were prepared from mouse spleens and stimulated with 0.5 μg/mL of different RBD proteins or equimolar amounts of RPMI 1640 culture medium (negative control) for 20 h. IFN-γ and IL-4-secreting cells were detected using ELISpot kits (MABTECH, Stockholm, Sweden) according to the manufacturer’s protocol, and spots were counted using an ELISpot Analyzer ImmunoSpot (CTL, USA).

### 2.9 Fluorescence-activated cell sorting

Lymphocyte sub-populations, including CD3^+^, CD3^+^CD4^+^, and CD3^+^CD8^+^ cells, were determined using fluorescence-activated cell sorting (FACS; BD FACS Canto™ II). IFN-γ and IL-4 levels were detected using a commercial kit (BioLegend, CA, USA) and analyzed using FACS (BD, USA). The following antibodies were used: CD4 monoclonal antibody (GK1.5, Super Bright™ 436; eBioscience™); CD8a monoclonal antibody (53-6.7, Alexa Fluor™ 488, eBioscience™); IL-4 monoclonal antibody (11B11, PE, eBioscience™); IFN gamma monoclonal antibody (XMG1.2, APC, eBioscience™).

### 2.10 Statistical analyses

Statistical analyses were conducted using GraphPad Prism software. The IC_50_ values were determined using a log (inhibitor) vs. response-variable slope (four parameters) test, whereas correlation analysis was conducted using linear regression (Pearson’s analysis). Multiple comparisons were performed using one- or two-way analysis of variance (ANOVA) and Tukey’s multiple comparison tests. P-values <0.05 were considered statistically significant.

## 3 Results

### 3.1 Antigen design and characterization

To facilitate the induction of long-term and high levels of broadly neutralizing antibody (bnAb) and cellular immune responses by our vaccine candidates, we designed several mRNA molecules based on the chimeric RBD of SARS-CoV-2 VOC, named RBD2.1, RBD2.2, RBD3, RBD3.U2, and RBD4. To induce bnAbs, we selected the RBD regions of different lineages of SARS-CoV-2 VOC as the RBD component of mRNA molecules representing key stages of SARS-CoV-2 evolution, and these RBDs were connect with GGGGS linker. These VOCs included the Wuhan-Hu-1 strain (D614G prototype) and the Beta (B.1.351), Delta (B.1.617.2), and Omicron (BA.4) variants. Considering the expression efficiency of mRNA *in vivo*, we designed different sequence lengths with 2, 3, or 4 RBD molecules. Additionally, the 5′-UTR of mRNA contains structural elements, which are recognized by cell-specific RNA binding proteins, thereby affecting the translation of the molecule (24). To enhance protein production, we also optimized a different 5′-UTR (5′-UTR-2) in the RBD3.U2 molecule. Finally, to further enhance the immunogenicity and extend antibody persistence, all mRNA molecules carried an Fc fragment at the end of the RBD. Additionally, YTE (Met252Tyr, Ser254Thr, and Thr256Glu) mutations were introduced in the Fc fragments of RBD2.2, RBD3, RBD3.U2, and RBD4 (25) (**Figure 1**). All mRNA molecules were encapsulated in LNPs, and the main quality parameters of the mRNA-LNPs were evaluated. The particle size, PDI, encapsulation efficiency, and integrity of mRNA-LNPs were 110 nm, 0.055, 95%, and 90%, respectively.

### 3.2 Elicitation of broad-neutralization activity in BALB/c mice

We analyzed the immunogenicity of these constructs in mice. All groups of mice were intramuscularly immunized with mRNA-LNPs twice on days 0 and 21. Among them, five groups were immunized with 5 μg of RBD2.1, RBD2.2, RBD3, RBD3.U2, and RBD4, respectively. To explore whether co-immunization with a combination of RBD components from two mRNA vaccines can induce stronger bnAb responses, one group of mice was intramuscularly immunized with a total of 5 μg of an RBD2.1 and RBD2.2 mixture at a 1:1 ratio. Mice in the control group received LNP alone. Mouse serum was collected to detect neutralizing antibodies on day 31 (**Figure 2A**).

To evaluate the ability of mRNA vaccines to induce a broadly neutralizing antibody activity, we evaluated the neutralization ability of sera to neutralize SARS-CoV-2 pseudoviruses of different lineages, including the D614G prototype, B.1.351, B.1.617.2, BA.4/5, BA.2.76, B.1.1.529, XBB.1, and BQ.1.1. All immunogens induced high titers of neutralizing antibodies against the pseudoviruses that belonged to the lineage covered by the RBD components in the constructs. However, none of the five constructs induced any neutralization activity against the epidemic variants XBB.1 and BQ.1.1. This suggested that RBD components of epidemic variants may be necessary for the production of bnAbs. Nevertheless, the serum neutralization antibodies induced by RBD4 against different pseudoviruses were at low levels, which may be attributed to the influence of sequence length on molecular expression. Notably, combined immunization with RBD2.1 and RBD2.2 induced higher levels of neutralizing antibodies against the D614G prototype, B.1.351, and B.1.617.2 pseudoviruses than those in the RBD2.1, RBD2.2, or RBD4 groups (**Figure 2B**). These results indicated that combined immunization may trigger stronger and broader neutralization activity. The neutralizing antibody titers of sera from the RBD3.U2 group were significantly higher than those of the RBD3 group, which may be related to the preferred 5′-UTR-2. Overall, the combination of two or three RBDs could trigger the production of high levels of neutralizing antibodies in mice. Additionally, 5′-UTR-2 enhanced the mRNA molecular immunogenicity, with the bivalent vaccines demonstrating the ability to increase the serum-neutralizing activity.

### 3.3 RBDco design and its immunogenicity in BALB/c mice

**Figure 3:**
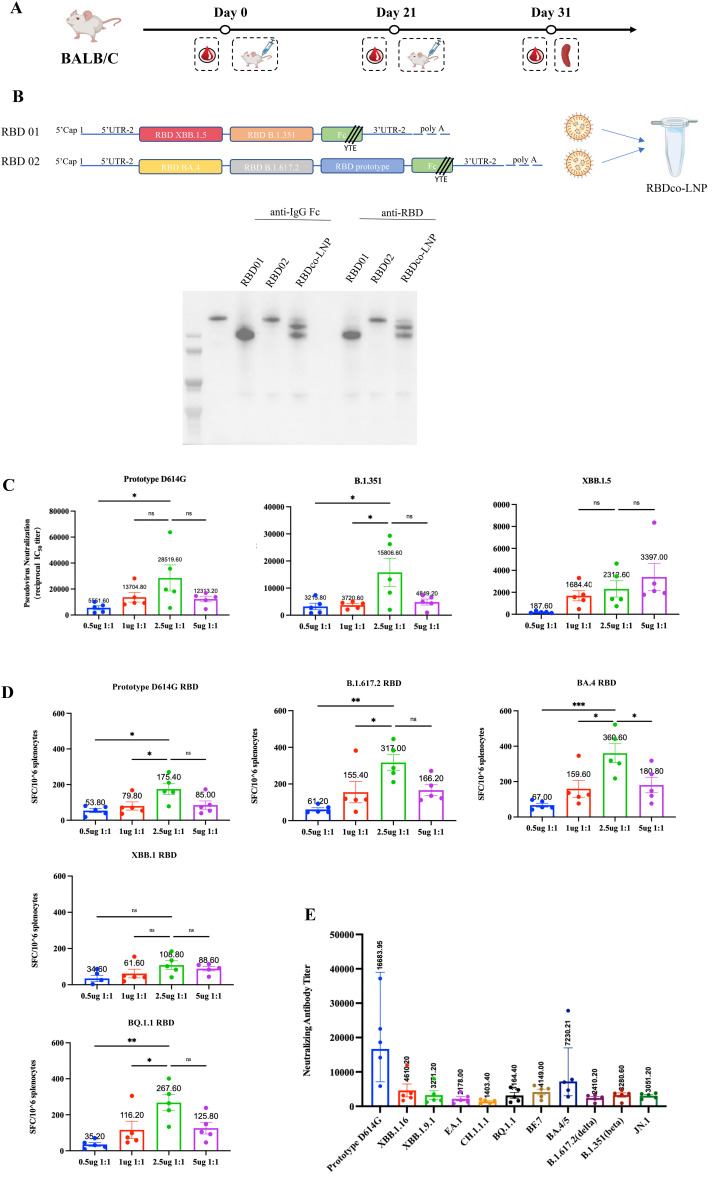
Efficacy and dosage of candidate bivalent vaccines. **(A)** Vaccination regimen for mice. Mice (n = 5) were randomly assigned to six groups and immunized with the respective immunogens twice at a 3-week interval. **(B)** Schematic diagram of vaccine preparation and expression in 293T cells. **(C)** Analysis of pseudovirus neutralization antibody titers in mouse sera from different immunization groups. Each antibody titer for an individual animal is indicated by a dot. **(D)** Analysis of cellular immune responses in mouse sera from different immunization groups. **(E)** Analysis of pseudovirus neutralization antibody titers in mouse sera from different immunization groups. ns, non-significant, *P < 0.05; **P < 0.01; ***P < 0.001.

Based on the abovementioned results, we designed a bivalent vaccine based on two and three combined chimeric RBDs named RBDco. To achieve a better neutralization response to epidemic strains, we introduced the RBD from the XBB.1.5 epidemic strain into the vaccine design. Specifically, both constructs contained the basic components of mRNA vaccines and 5′-UTR-2. Both constructs had Fc fragments with YTE mutations at the 3′-end. RBD01 contained RBDs from the XBB.1.5 and B.1.351 variants, whereas RBD02 contained RBDs from the D614G prototype and B.1.617.2 and BA.4 variants. These two constructs were encapsulated in LNPs at a 1:1 ratio. Subsequently, RBD01 and RBD02 mRNA stock solutions were transfected into 293T cells. Simultaneously, RBDco-LNP samples were directly transfected into 293T cells to detect the *in vitro* expression activity of RBDco. Western blot analysis showed that both RBD01 and RBD02 were successfully expressed in 293T cells at the expected protein size. RBDco-LNP was also expressed in 293T cells, with the two components showing similar expression effects (**Figure 3B**). To assess immunogenicity, four groups of mice (n = 10) were intramuscularly immunized twice on days 0 and 21 with a total dose of 0.5, 1, 2.5, and 5 μg of RBDco, respectively (**Figure 3A**). Mouse serum samples were collected for detecting neutralizing antibodies on day 31. All different doses of RBDco showed neutralization activity against the D614G prototype, B.1.351, and XBB.1.5 pseudoviruses. Among them, the neutralization antibody titers of sera from the 2.5 μg group against the D614G prototype, B.1.351, and XBB.1.5 pseudoviruses were 28519, 15806, and 2312, respectively. In terms of dose response, the 2.5 μg group induced significantly higher neutralizing antibody levels against the D614G prototype and B.1.351 compared with the levels in other groups, whereas sera from the 5 μg group were the most effective in neutralizing against XBB.1.5 (**Figure 3C**).

We also determined the cell-mediated immunity in mice immunized with RBDco by using an IFN-γ ELISpot assay on isolated spleen cells re-stimulated with different RBD proteins from VOC. RBDco induced a strong cellular immune response against different RBDs, especially those of the XBB.1 and BQ.1.1 epidemic variants. Cells in the 2.5 μg group exhibited the highest level of cellular immune response against all RBD stimuli (**Figure 3D**).

To determine whether the 2.5 μg group could trigger a broad-neutralization activity, we evaluated the neutralization activity of sera from the 2.5 μg group against 10 key pseudoviruses, which originated from different lineages of viral evolution and potential epidemic variants. The neutralizing antibody titers against different pseudoviruses were all above 1000. The neutralizing antibody titers against the XBB.1.16, XBB.1.9.1, EA.1, CH.1.1.1 and JN.1 pseudoviruses reached 4610, 3274, 2476, 1403 and 3051 respectively (**Figure 3E**). Based on these results, we concluded that RBDco induced a broad-neutralization activity, with the optimal injection dose in mice being 2.5 μg.

### 3.4 Protection of RBDco against novel coronavirus variant (Omicron-XBB.1.16) challenge

**Figure 4:**
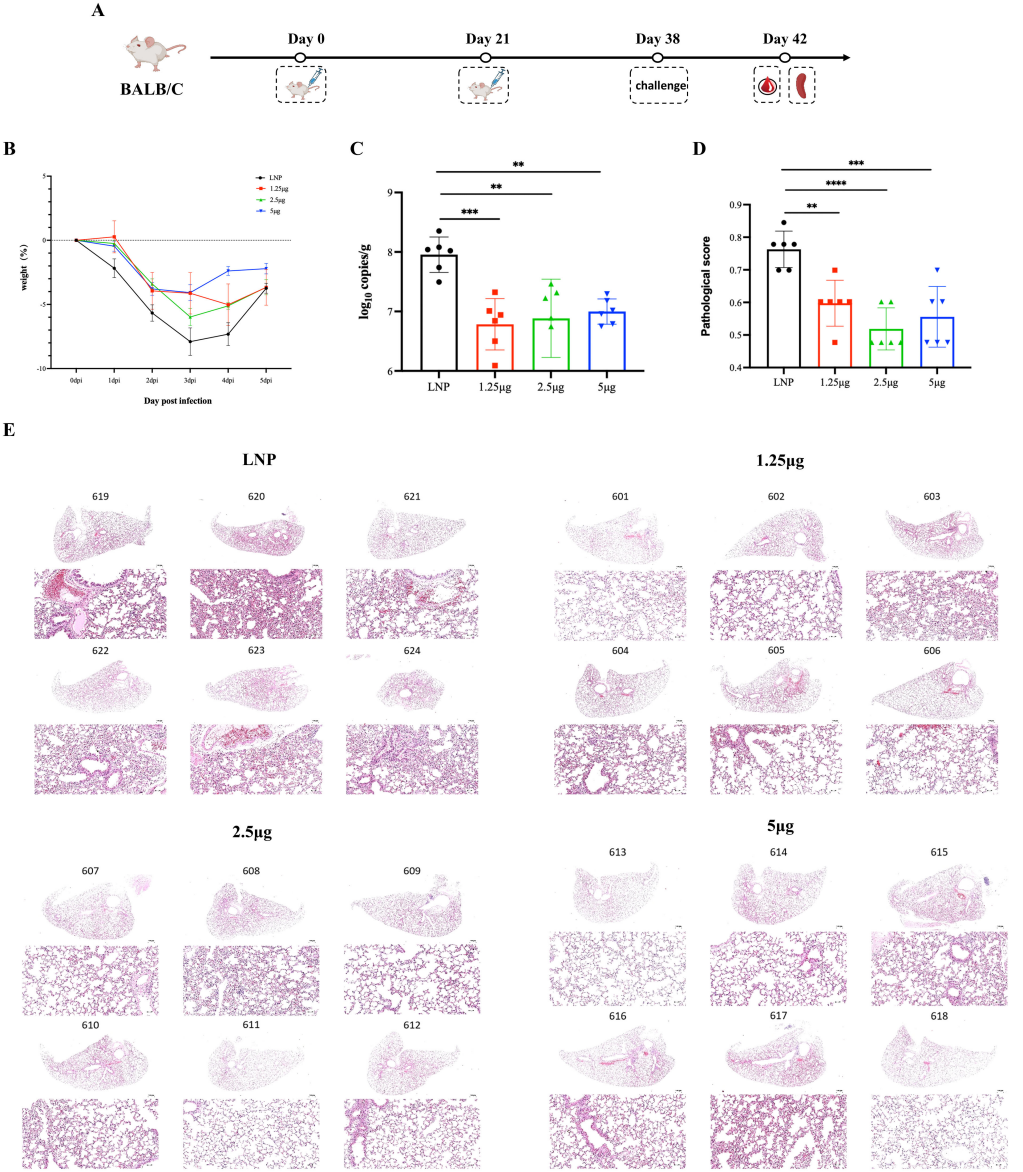
Protective effect of RBDco against a novel coronavirus variant (Omicron-XBB. 1.16) infection. **(A)** Nasal drops were used for the challenge. All experimental animals received Omicron (XBB. 1.16; virus batch number 23V08P, original titer of 1 × 10^7^ TCID_50_/mL) via nasal drip (100 μL/mouse) at a dose of 1 × 10^6^ TCID_50_ per mouse. **(B)** Assessment of body weight and body temperature. **(C)** Sample processing: On the fifth day after the challenge, mice were euthanized, and their lungs were dissected to observe general pathological changes. RNA from the lungs was used in qRT-PCR assays (one-step method) to detect the viral load. **(D, E)** After euthanasia, the left lung lobe was collected and fixed in formalin, and then stained with HE for disease observation. The pathological photos of each mouse's lung tissue pathology were rated, and the scoring was mainly aimed at the main pathological characteristics of COVID-19. **P < 0.01; ***P < 0.001; ****P < 0.0001.

To further demonstrate the viral clearance ability of RBDco, we evaluated its protection against an epidemic variant (Omicron-XBB.1.16) challenge in vaccine-immunized BALB/c mice (**Figure 4A**). We evaluated the body weight, lung viral load, and lung lesions of mice after the challenge. In particular, the body weight of mice in the RBDco immune group decreased (with a 2.21% for the 5 μg compared with that at 0 dpi) and was significantly lower than that in the control group (decreased by 3.74%). This decrease was dose-dependent (**Figure 4B**). The average viral loads in the lung tissue of the RBDco immune group were significantly lower than those in the control group. Murine lungs showed high viral loads in the control group, with an average load of 7.95 log copies/g. The average viral loads in the lung tissue of mice in the 1.25, 2.5, and 5 μg groups were 6.78, 6.88, and 7.00 log copies/g, respectively (**Figure 4C**). The comprehensive score of lung tissue pathological damage in the RBDco immune group of mice was significantly reduced compared with that in the control group (**Figures 4D, E**). These results indicated that the RBDco effectively reduced the lung tissue pathological damage induced by the XBB.1.16 challenge.

### 3.5 RBDco induced high levels of humoral immunity in crab-eating macaques

**Figure 5:**
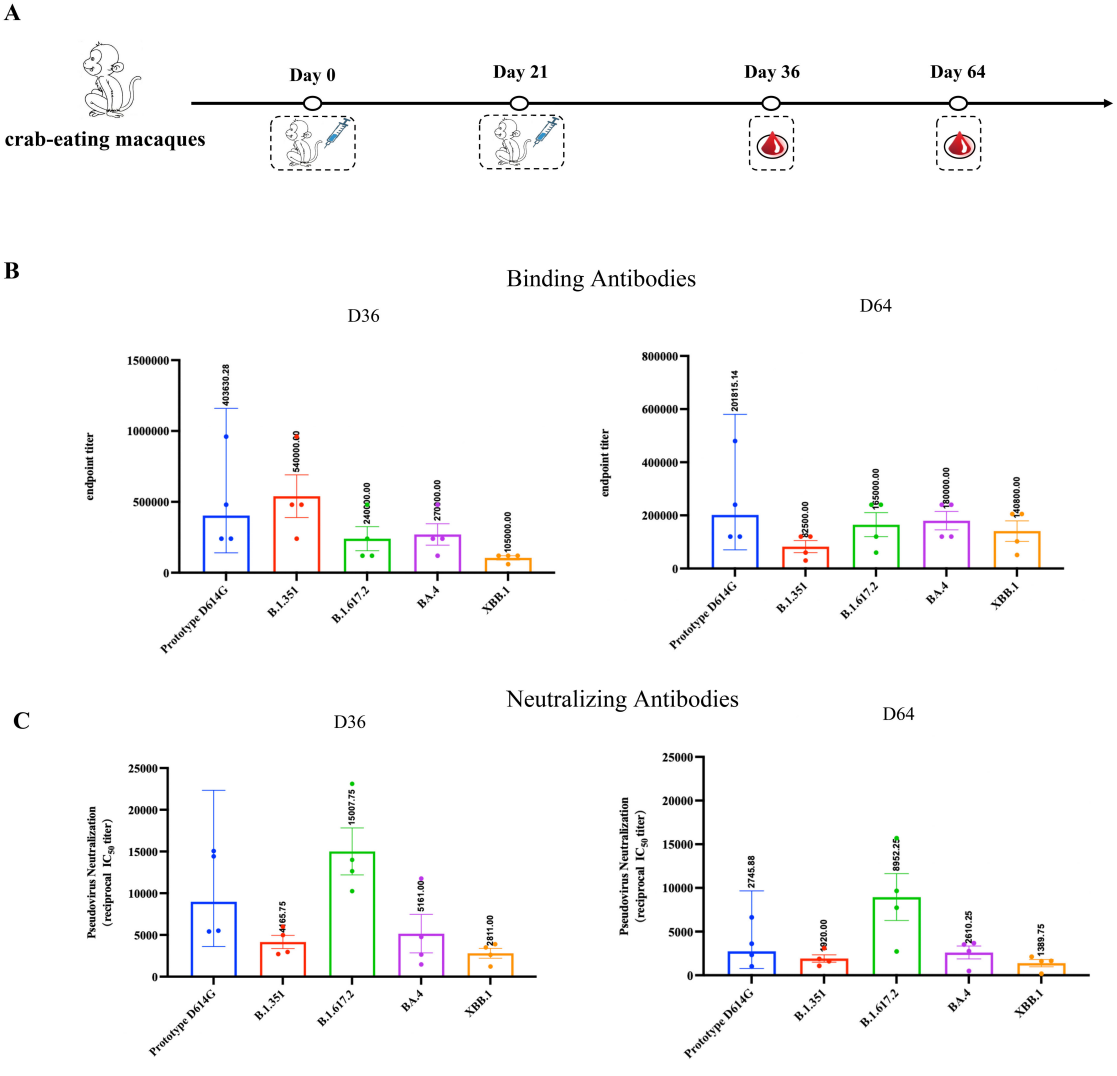
RBDco induces high levels of humoral immunity in crab-eating macaques. **(A)** Vaccination regimen for crab-eating macaques. For the vaccination of non-human primates, cynomolgus monkeys were immunized twice on D0 and D21. Subsequently, the levels of binding **(B)** and neutralizing **(C)** antibodies against different strains of SARS-CoV-2 in their sera were detected on D36 and D64. Sera from mice or cynomolgus monkeys were collected and inactivated at 56°C for 0.5 h before measuring SARS-CoV-2-specific IgG and neutralizing antibodies.

To further demonstrate the effectiveness of RBDco in non-human primates, we immunized cynomolgus monkeys (n = 4) twice, on days 0 and 21, and detected the levels of binding and neutralizing antibodies in their sera on days 36 and 64 (**Figure 5A**). RBDco induced high levels of binding antibody titers in cynomolgus monkeys after two immunizations. The specific binding antibody titers against the D614G prototype, B.1.351, and the XBB.1 epidemic variant reached 403630, 540000, and 105000, respectively, on day 36. Additionally, RBDco vaccination induced strong neutralizing activities against SARS-CoV-2 pseudoviruses. The neutralizing antibody titers against B.1.617.2 and the XBB.1 epidemic variant were 15007 and 2811, respectively. We also assessed the binding antibody and neutralizing antibody levels on day 64 in crab-eating macaques. The XBB.1-specific binding antibody titer reached 140800, whereas the neutralizing antibody titer was 1389 (**Figures 5B, C**). These results indicated that RBDco can induce high levels of humoral immunity in non-human primates.

### 3.6 Sequential immunization with inactivated vaccines and RBDco induced a Th1-biased response

**Figure 6:**
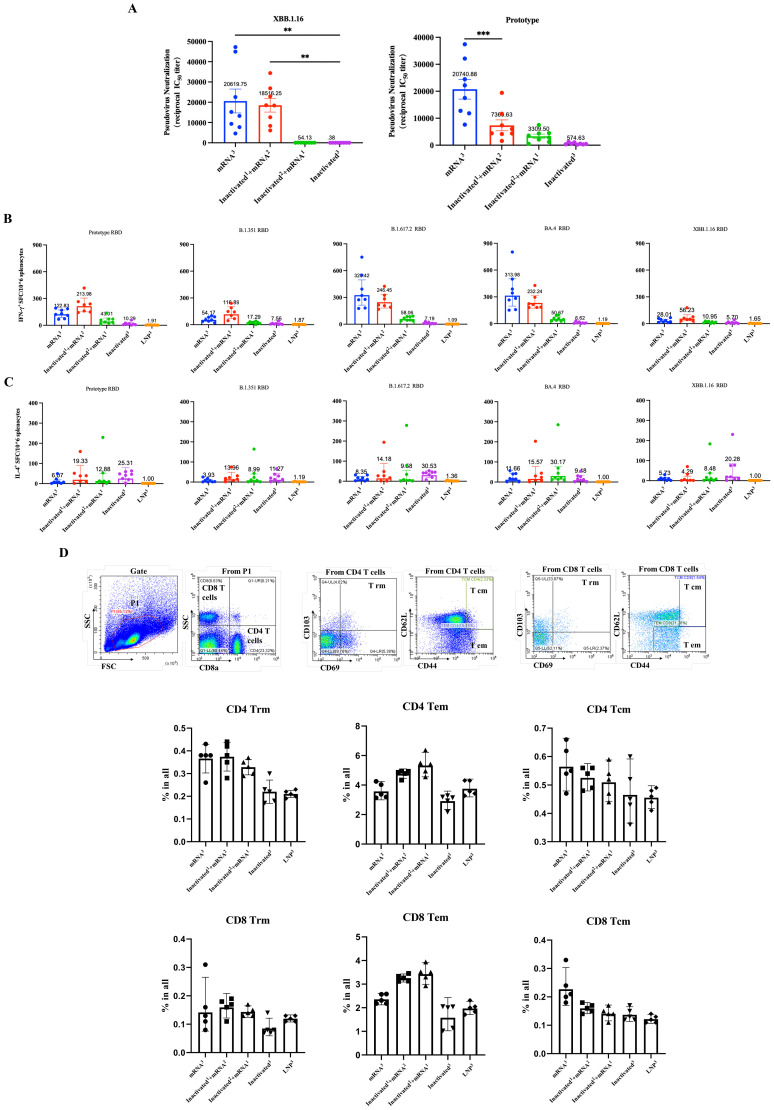
Effect of sequential immunization on the production of neutralization antibodies and cytokines. **(A)** Analysis of pseudovirus neutralization antibody titers in mouse sera from different sequential immunization groups. **(B)** IFN-γ secretion levels from different sequential immunization groups. **(C)** IL-4 secretion levels from different sequential immunization groups. Each antibody titer for an individual animal is represented by a dot. ** P < 0.01; *** P < 0.001. **(D)** Flow cytometry was used to detect the proportions of CD4^+^ and CD8^+^ Trm, Tem, and Tcm cells in mouse spleens 10 d after the final immunization.

Considering the wide implementation of SARS-CoV-2 inactivated vaccines, evaluating the potential application of novel vaccines as heterologous booster shots is necessary. To simulate the current vaccination scenario in the population, we immunized mice (n = 8) with one or two doses of inactivated vaccines and then boosted them with RBDco. Two groups of mice were immunized thrice with RBDco or an inactivated vaccine. Mice in the control group were immunized thrice with LNPs (**Table 1**). Sera were collected 10 d after the final immunization. Notably, the neutralizing antibody titers were improved in RBDco-boosted groups compared with those in the inactivated^3^ group. The neutralizing antibody titers against the D614G prototype and XBB.1.16 pseudoviruses in the inactivated^1^ + mRNA^2^ group were higher than those in the inactivated^2^ + mRNA^1^ group. Similarly, neutralizing antibody titers against XBB.1.16 and the D614G prototype in the mRNA^3^ group were significantly higher than those in the inactivated^1^ + mRNA^2^ group (**Figure 6A**).

**Table 1:** Sequential immunization strategy.

Group	Day 0	Day 21	Day 42
mRNA^3^	RBDco	RBDco	RBDco
Inactivated^1^ + mRNA^3^	Inactivated vaccine	RBDco	RBDco
Inactivated^2^ + mRNA^1^	Inactivated vaccine	Inactivated vaccine	RBDco
Inactivated^3^	Inactivated vaccine	Inactivated vaccine	Inactivated vaccine
LNP^3^	LNP	LNP	LNP

We further examined the cellular immune responses. We collected mice spleen cells and detected the levels of secretion of IFN-γ and IL-4. All vaccinated groups, except the inactivated^3^ group, induced high levels of IFN-γ secretion, accompanied by low levels of IL-4 secretion when stimulated by the D614G prototype, B.1.315, B.1.617.2, BA.4, and XBB.1.16 RBD proteins. Conversely, mice in the inactivated^3^ group secreted slightly higher levels of IL-4 than those of IFN-γ. Notably, mice in the mRNA^3^ and inactivated^1^ + mRNA^2^ groups secreted slightly higher levels of IFN-γ than those in the other groups (**Figures 6B, C**). Overall, boosting with RBDco altered T-helper cell bias from Th2, which is induced by inactivated vaccines, to Th1. These results indicated that sequential immunization with RBDco can enhance cellular immune responses in mice.

Finally, we investigated the enhancing effect of sequential immunity on memory T-cell differentiation. We used flow cytometry to detect the proportions of CD4^+^ and CD8^+^ Trm, Tem, and Tcm cells in mouse spleen cells. The proportions of CD4^+^ and CD8^+^ Trm cells in the inactivated^1^ + mRNA^2^ group were slightly higher than those in the other groups. Similarly, the proportions of CD4^+^ and CD8^+^ Tem cells in the inactivated^2^ + mRNA^1^ were slightly higher than those in the other groups (**Figure 6D**). These results indicated that sequential immunization with the inactivated vaccine and RBDco can promote the differentiation of memory T-cells.

### 3.7 Effect of the immunization interval on neutralizing antibodies and antibody persistence

**Figure 7:**
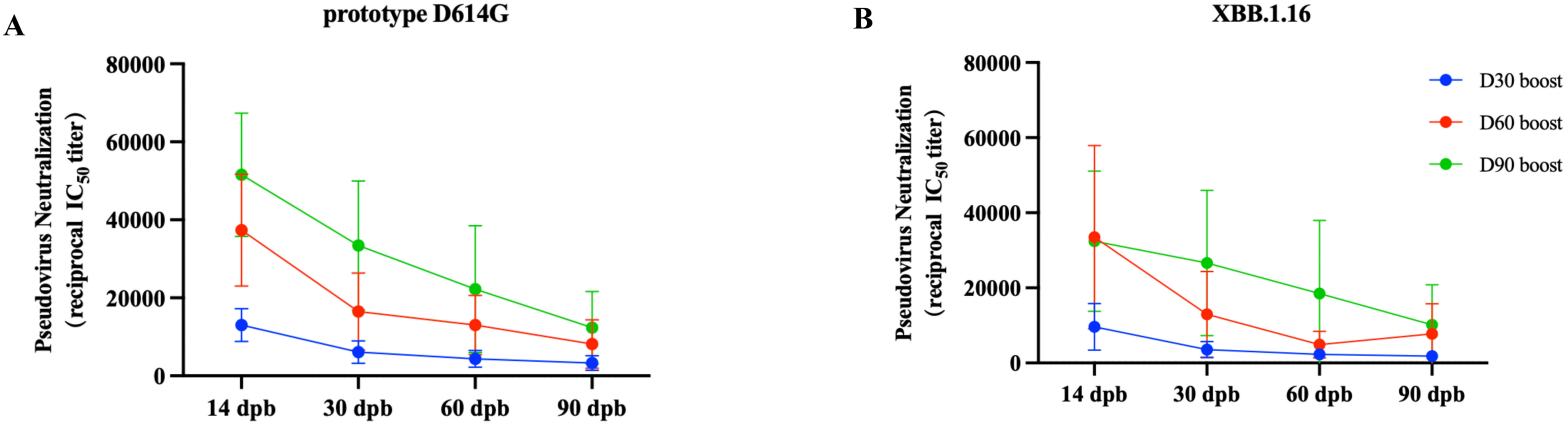
Effect of the immunization interval on neutralizing antibodies and antibody persistence. Vaccination regimen for mice. Mice (n = 10) were randomly assigned to four groups, immunized once on D0, and then received booster immunizations on D30, D60, or D90. The pseudovirus neutralization antibody titers in mouse sera from different encapsulation procedure groups were analyzed. **(A)** Prototype D614G pseudovirus. **(B)** XBB.1.16 pseudovirus.

To achieve high immunogenicity and persistence, we introduced YTE mutations in the Fc fragment carried by the RBDco to prolong the half-life of the expressed antibodies. In addition, the immune interval may also impact the persistence of antibodies. Therefore, three groups of mice (n = 10) were immunized with RBDco at day 0 and boosted at days 30, 60, and 90, respectively. The levels of neutralizing antibodies against the D614G prototype and XBB.1.16 pseudoviruses were detected on days 14, 30, 60, and 90 after booster vaccination. The level of neutralizing antibodies in mice was significantly enhanced with increasing immunization intervals. The neutralizing antibody titer against the D614G prototype in the day-90 booster group was four times higher than that in the day-30 booster group and 1.3 times higher than that in the day-60 booster group (**Figure 7A**). The neutralizing antibody titers of the day-60 and day-90 booster groups against the XBB.1.16 epidemic strain were both 3.5 times higher than those in the day-30 booster group (**Figure 7B**). These results indicated that an increase in the immunization interval could effectively enhance the RBDco-induced neutralizing antibody titers in mice. Furthermore, we explored the persistence of neutralizing antibodies in the day-90 booster group. The level of neutralizing antibodies against the D614G prototype was three times lower at 90 d compared with that at 14 d after secondary immunization. The level of neutralizing antibodies against the XBB.1.16 epidemic strain was two times lower at 90 d compared with that at 14 d after secondary immunization. These results indicated that RBDco-induced antibodies had excellent persistence.

## 4 Discussion

In this study, we established an mRNA vaccine candidate, RBDco, based on the chimeric design of RBD sequences from different SARS-CoV-2 VOCs of different lineages to improve the production of broadly neutralizing antibodies against SARS-CoV-2. The vaccine candidate effectively induced neutralizing antibodies in both mice and non-human primates against several pseudoviruses of different lineages, including the possible epidemic variants XBB.1, XBB.1.9.1, and EA.1. Simultaneously, RBDco could reduce lung tissue pathological damage when challenged with XBB.1.16. The sequential immunization of RBDco in pre-immunized mice vaccinated with inactivated vaccines effectively improved both broadly neutralizing antibody and cellular immune responses.

To date, numerous studies on COVID-19 vaccines have focused on improving the broad-neutralization activities of vaccines. Zhang et al. reported an LNP-encapsulated SARS-CoV-2 RBD-based mRNA vaccine that could induce bnAbs and Th1-biased cellular immune responses (26). Yang et al. demonstrated that a disulfide-linked SARS-CoV-2 RBD dimer could induce higher nAb titers than an RBD monomer (27). To curb the rapidly mutated SARS-CoV-2 virus, COVID-19 vaccines are expected to induce bnAbs, as bnAbs are one of the most effective means of inhibiting viral transmission (28). Moreover, bnAbs can kill infected cells and prevent the spread of viruses between cells (29). Some vaccines can induce antibodies against the Wuhan and Beta variants but are powerless against the emerging Delta and Omicron variants (5–7). Structural analyses of antibodies isolated from the sera of convalescent patients have revealed that bnAbs need to undergo complex antibody affinity maturation and somatic mutation processes (30), with long CDR3 regions or map to more conserved but less accessible regions of sarbecovirus RBDs (31, 32). Generating RBD mosaics is also a strategy used to enhance broad specificity (33), as it can transform the immune subdominant RBD epitopes into immune dominant epitopes (34). Studies have reported that in RBD mosaic or booster immune vaccine strategies, selecting VOCs at appropriate distances in the evolutionary lineage of the virus as antigens can effectively induce bnAbs (35, 36). More importantly, the RBD mosaic design can effectively induce the differentiation of B-cells, producing cross-reactivity and generating high-affinity antibodies targeting conserved viral epitopes (37). Other highly mutated virus vaccine studies demonstrated that vaccines targeting a single viral target site are prone to producing drug-resistant strains, whereas simultaneously targeting multiple sites is beneficial for the production of vaccines with broad specificity (38–40). To target the diverse and frequent mutation of the SARS-CoV-2 virus, RBDco, a bivalent vaccine, was designed to contain chimeric RBDs. An attempt was made to introduce different lineages of SARS-CoV-2 VOC RBDs to induce bnAbs. Furthermore, the RBDco design allows the possibility of replacing the RBD sequences, thus better responding to future emerging variants. The RBDco-induced neutralizing antibodies showed high levels of neutralizing efficacy against 11 variants, including prototype and circulating variants, in mice.

To better predict the efficacy and safety of human vaccines before clinical trials, evaluating these components in non-human primates is crucial (41–43). We tested the humoral immune responses induced by RBDco in non-human primates and found that RBDco induced high levels of humoral immune responses, indicating the feasibility of its application in clinical trials.

Approximately all individuals worldwide have been exposed to COVID-19. A new COVID-19 vaccine should reactivate the immune response in pre-immunized individuals. In certain parts of the world, where the majority of the population is vaccinated with COVID-19-inactivated vaccines, the immune enhancement effect of a novel vaccine is particularly important (44, 45). Thus, we performed sequential immunization experiments using inactivated vaccines and RBDco to evaluate the immune enhancement efficacy of RBDco. The sequential immunization strategy improved the immunogenicity of RBDco in humoral and cellular immune responses. To further improve vaccine immunogenicity, distinct mRNA or other types of vaccines can be added to the immunization process (46, 47). Repeated infection with SARS-CoV-2 is an important issue to be addressed for the development of new COVID-19 vaccines. Memory T-cells can reactivate and clear the virus during secondary infection. Therefore, vaccines that can effectively induce memory T-cell differentiation are promising in the fight against secondary SARS-CoV-2 infections (48–50). We detected changes in CD4 and CD8 Tcm cells after sequential immunization with inactivated vaccines and RBDco. We showed that sequential immunization significantly increased the proportion of Tcm cells at a level higher than that in the group treated with a combination of all-mRNA vaccines and inactivated virus vaccines. These results indicated that sequential immunization significantly enhanced the activation of memory T-cells.

Currently, achieving the global vaccination of all individuals with the COVID-19 vaccine is difficult; therefore, the sustainability of antibodies produced by COVID-19 vaccines is important (51, 52). The Fc fragment, as an immune enhancer, can also help to enhance broad antibody responses (53). Because the FcRn of the Fc fragment is expressed on the surface of various immune cells, including NK cells, macrophages, and neutrophils, it helps activate the innate immune response (54, 55). The YTE mutation in the Fc fragment can prolong the half-life of the Fc fusion protein. Considering the rapid transmission of SARS-CoV-2 via the mucosal route, antibodies with longer half-lives on mucosal tissues could enhance the mucosal immune response (56, 57). According to current mRNA vaccine research, the titers of neutralizing antibodies generally reach their highest level of 7–14 d after booster immunization and gradually decrease thereafter (58–60). Most studies have shown a decrease in the antibody level of more than ten times between 30 and 60 d. In our study, RBDco expressed the Fc terminal carrying a YTE mutation, which has been reported to effectively increase the half-life of the protein. We tested the persistence of RBDco-induced antibodies and also evaluated the effect of the booster immunization interval on antibody titers. The antibody levels in mice were only decreased 2–3 fold after 90 d compared with those at 14 d after booster immunization. In particular, antibody titers were maintained at a high level for a 90-d interval, contributing to immunity against the virus. Thus, the Fc co-expression and YTE mutation design is a feasible method for extending the protein half-life and thereby prolonging antibody persistence. At present, we are continuing to carry out RBDco neutralization research against the latest potential epidemic variants. We are also actively preparing for the industrialization of RBDco and conducting in-depth toxicology and pharmacology research.

SARS-CoV-2 may evolve into a seasonal epidemic virus similar to influenza. Based on predictions of viral mutations, investigating vaccines that can respond quickly to VOC and induce bnAbs is important. The chimeric RBD bivalent vaccine RBDco used in this study induced high levels of bnAbs. Owing to its potentially replaceable RBD element, this vaccine can respond faster to viral mutations and serve as a candidate for future broad-neutralization COVID-19 vaccines.

## Data Availability

The original contributions presented in the study are included in the article/supplementary material. Further inquiries can be directed to the corresponding authors.
